# RxNorm for drug name normalization: a case study of prescription opioids in the FDA adverse events reporting system

**DOI:** 10.3389/fbinf.2023.1328613

**Published:** 2024-01-05

**Authors:** Huyen Le, Ru Chen, Stephen Harris, Hong Fang, Beverly Lyn-Cook, Huixiao Hong, Weigong Ge, Paul Rogers, Weida Tong, Wen Zou

**Affiliations:** ^1^ Division of Bioinformatics and Biostatistics, Jefferson, AR, United States; ^2^ Office of Translational Science, Center for Drug Evaluation and Research, U.S. Food and Drug Administration, Silver Spring, MD, United States; ^3^ Office of Scientific Coordination, Jefferson, AR, United States; ^4^ Division of Biochemistry Toxicity, National Center for Toxicological Research, U.S. Food and Drug Administration, Jefferson, AR, United States

**Keywords:** RxNorm, RxCUI, FAERS, drug name normalization, prescription opioids, drug safety

## Abstract

Numerous studies have been conducted on the US Food and Drug Administration (FDA) Adverse Events Reporting System (FAERS) database to assess post-marketing reporting rates for drug safety review and risk assessment. However, the drug names in the adverse event (AE) reports from FAERS were heterogeneous due to a lack of uniformity of information submitted mandatorily by pharmaceutical companies and voluntarily by patients, healthcare professionals, and the public. Studies using FAERS and other spontaneous reporting AEs database without drug name normalization may encounter incomplete collection of AE reports from non-standard drug names and the accuracies of the results might be impacted. In this study, we demonstrated applicability of RxNorm, developed by the National Library of Medicine, for drug name normalization in FAERS. Using prescription opioids as a case study, we used RxNorm application program interface (API) to map all FDA-approved prescription opioids described in FAERS AE reports to their equivalent RxNorm Concept Unique Identifiers (RxCUIs) and RxNorm names. The different names of the opioids were then extracted, and their usage frequencies were calculated in collection of more than 14.9 million AE reports for 13 FDA-approved prescription opioid classes, reported over 17 years. The results showed that a significant number of different names were consistently used for opioids in FAERS reports, with 2,086 different names (out of 7,892) used at least three times and 842 different names used at least ten times for each of the 92 RxNorm names of FDA-approved opioids. Our method of using RxNorm API mapping was confirmed to be efficient and accurate and capable of reducing the heterogeneity of prescription opioid names significantly in the AE reports in FAERS; meanwhile, it is expected to have a broad application to different sets of drug names from any database where drug names are diverse and unnormalized. It is expected to be able to automatically standardize and link different representations of the same drugs to build an intact and high-quality database for diverse research, particularly postmarketing data analysis in pharmacovigilance initiatives.

## 1 Introduction

Spontaneous AE reporting systems are essential components in post-marketing drug surveillance to assess human drug safety. They are an important source of information for the detection of previously unidentified or unknown AEs in clinical trials, and are important for real-world safety assessments in various populations and clinical practice (Noguchi et al., 2021). FAERS is a database for post-marketing drug safety monitoring and was constructed for the pharmaceutical industry, healthcare providers and consumers to easily report human adverse events to the FDA. Since 1969 into the present, FAERS has been the largest repository of spontaneously reported AEs maintained by any single country, with over 16 million AE reports (Administration, USFDA, 2021). Therefore, numerous studies have used this publicly available database to assess post-marketing drug safety and risks (Winnenburg et al., 2015; Botsis et al., 2017; Fukazawa et al., 2018; Ly et al., 2018; Schotland et al., 2018) to support the FDA’s post-marketing safety surveillance program for currently marketed US drugs and therapeutic biologics products (Rodriguez et al., 2001; Trontell, 2001; Wysowski and Swartz, 2005).

However, when mining adverse event reports from FAERS, it was noticed that although adverse events were coded as Preferred Terms (PTs) in MedDRA (Medical Dictionary for Regulatory Activities. Available at: http://www.meddra.org/), drug names in FAERS reports might appear different for various reasons including that both brand names and generic names of drugs are used; data are collected from various sources worldwide; discrepancies occur between the reported name and the correct name due to errors or misspellings when entering drug names to the system; drug names can change over time due to factors such as rebranding, patent expiration, or regulatory decisions; use of slang terms for drugs. Therefore, different formats of drug names for the same drug entity are present in the FAERS database for numerous drugs. Currently, there are no available tools to automatically group all the different drug name formats into a single drug entity without proper data pre-processing. Consequently, to ensure accurate analysis and interpretation of FAERS data, it is essential to consider all potential variations of drug names and reconcile them appropriately. Otherwise, it may result in incomplete data collection of adverse event reports from FAERS consequently leading to inaccurate results and interpretation. This phenomenon not only exists in FAERS, but also occurs in any other spontaneous reporting databases where drug names were diverse and unnormalized due to the unstructured entry (e.g., Yellow Card Scheme (Report a problem with a medicine or medical device. Available at: https://www.gov.uk/report-problem-medicine-medical-device)).

To the best of our knowledge, only a few studies have been conducted on the issue of drug name variations in FAERS reports. Some studies used the RxNorm for drug name mapping to try to build a standardized dataset of FAERS adverse events (Poluzzi et al., 2012; Banda et al., 2016; Khaleel et al., 2022), but detailed information on how the RxNorm mapping facilitated drug normalization was not provided. RxNorm is freely available and public-accessible, and has been developed by the National Library of Medicine and provides a standardized nomenclature for clinical drugs as well as the relationships of different names based on different external source vocabularies (Liu et al., 2005; Nelson et al., 2011). Evaluations of RxNorm for representative ambulatory electronic prescriptions (e-prescriptions) have shown that the coverage rate of RxNorm for representing clinical drugs is very high, up to 99.995%, in different random samples of thousands of e-prescriptions (O'Neill and Bell, 2010; Dhavle et al., 2016). In addition to searching the RxNorm concepts (i.e., standardized drug names) by exact match or normalized string search, RxNorm allows approximate matching to determine the most closely-related searching string-matched RxNorm concepts by fixing spelling errors as well as informal abbreviations and acronyms. Consequently, it might be effective to use RxNorm to automatically map heterogeneous drug names to a standard drug name to build up a clean and accurate dataset on FAERS-related studies.

In this study, a novel method was developed to use RxNorm for drug normalization in spontaneous reporting databases through the RxNorm API (https://rxnav.nlm.nih.gov/RxNormAPIs.html), which is a web interface for inputting and accessing information from the RxNorm data set. We have described each step of this process in detail through a case study involving retrieving FDA-approved opioid-associated adverse event reports. The effectiveness of our method using RxNorm application was demonstrated as capable of reducing the heterogeneity of opioid names entered by the public in the FAERS database, and therefore was able to build up a more complete dataset of opioid-related adverse event reports. This RxNorm method is not only applicable to FAERS but can also be useful with other spontaneous reporting databases where there is no fixed rule for drug name entries. The application of this method for drug normalization is expected to not only reduce the heterogeneities of drug name entries to overcome drug name discrepancies and inconsistencies; but also standardize the representation of drug-related adverse events or other data in a consistent and comparable manner. In addition, this RxNorm-driven method could facilitate the integration of data from multiple databases, technologies, or modalities to enhance the comprehensive analysis of drug-related data, leading to more robust insights and discoveries in drug safety and pharmacovigilance.

## 2 Materials and methods

**FIGURE 1:**
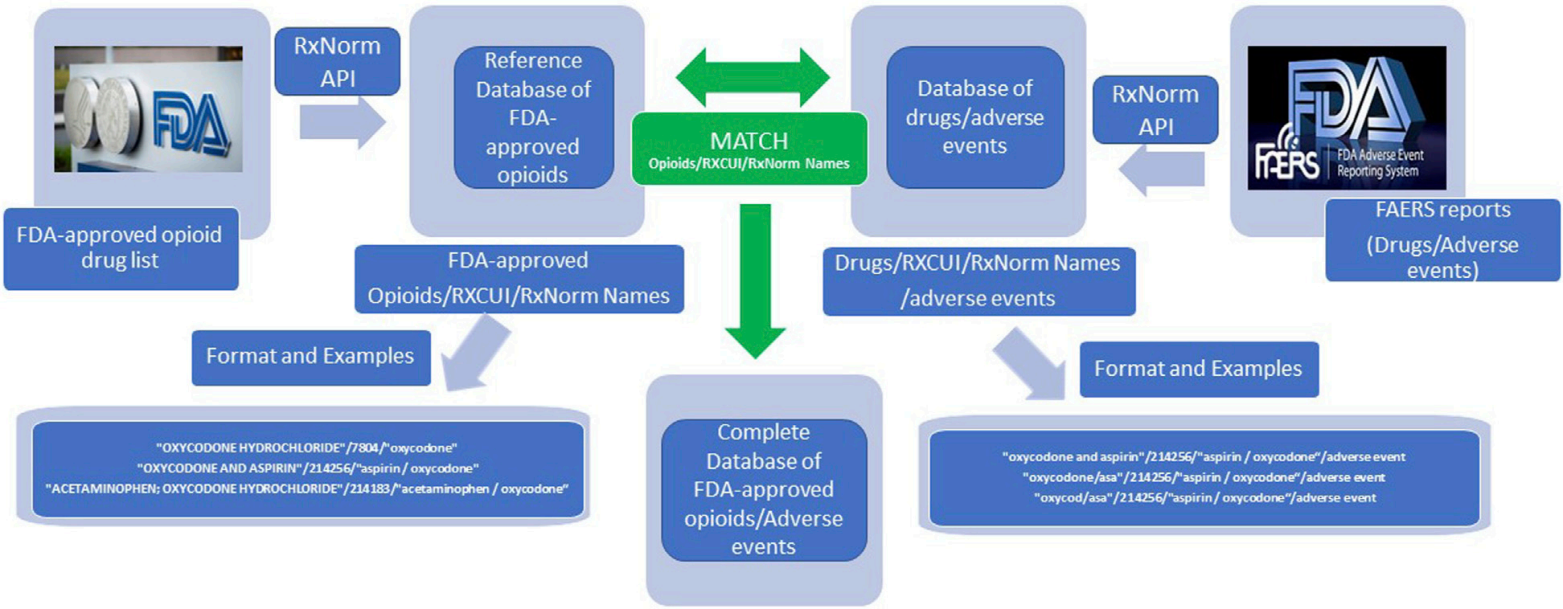
RxNorm API for drug name normalization workflow.

As shown in Figure 1, the entire list of FDA-approved drugs was downloaded in February 2021 (Drug Approvals and Databases. Available at: https://www.fda.gov/drugs/development-approval-process-drugs/drug-approvals-and-databases). Opioid names were manually identified from this list. The RxNorm API was then applied to map all FDA-approved opioids to their equivalent RxCUIs and RxNorm names. Each opioid name was associated with one RxCUI (an integer number), and one RxNorm name (a standardized drug name). RxCUI is the key component of RxNorm and is used as a unique, explicit identifier for an individual drug entity in RxNorm.

The adverse event reports (AERs) collected from FAERS between 2004 Quarter 1 and 2020 Quarter 3 were retrieved and downloaded in “xml” format (Administration, USFDA, 2021). Although drug names appear heterogeneous, they were located in “xml” tag pairs as <medicinalproduct></medicinalproduct> in AERs. The drug names were then automatically extracted from the reports and their usage frequencies were counted, respectively. The RxNorm API was applied to the extracted drug names in the same way to map all drug names to their respective RxCUIs and RxNorm names. The drug names were then clarified by matching their RxCUIs with the RxCUI list of the FDA-approved opioids in the database.

When using RxNorm API to find the matching RxCUI of a drug, a list of RxCUIs, instead of only one RxCUI, might be shown in the results. Therefore, their respective ranking scores represented how close the RxNorm concepts matched with the search strings. Under these circumstances, only the RxCUIs with the highest-ranking scores were accepted. To ensure the accuracy and avoid possible errors made by the algorithm in RxNorm API, manual checking was also applied to remove easily recognized mistakes in the RxCUI list of the FDA-approved opioids.

## 3 Results and discussion

### 3.1 Construction of a reference database for FDA-approved prescription opioids

The entire list of FDA-approved drugs as of February 2021 contained 7,436 drug names. After manual identification and RxNorm API application, a preliminary database was constructed that contained the information for 92 FDA-approved prescription opioids, their corresponding RxCUIs and RxNorm names, which we designated as the reference database for drug name normalization of FDA-approved prescription drugs in this study. Therefore, RxCUI/RxNorm should be an appropriate identifier or index for drug annotation and normalization. In addition, the application of RxCUI/RxNorm to the database makes it possible and available to relate all things associated with a specific drug.

### 3.2 A case study using prescription opioids in FAERS

**FIGURE 2:**
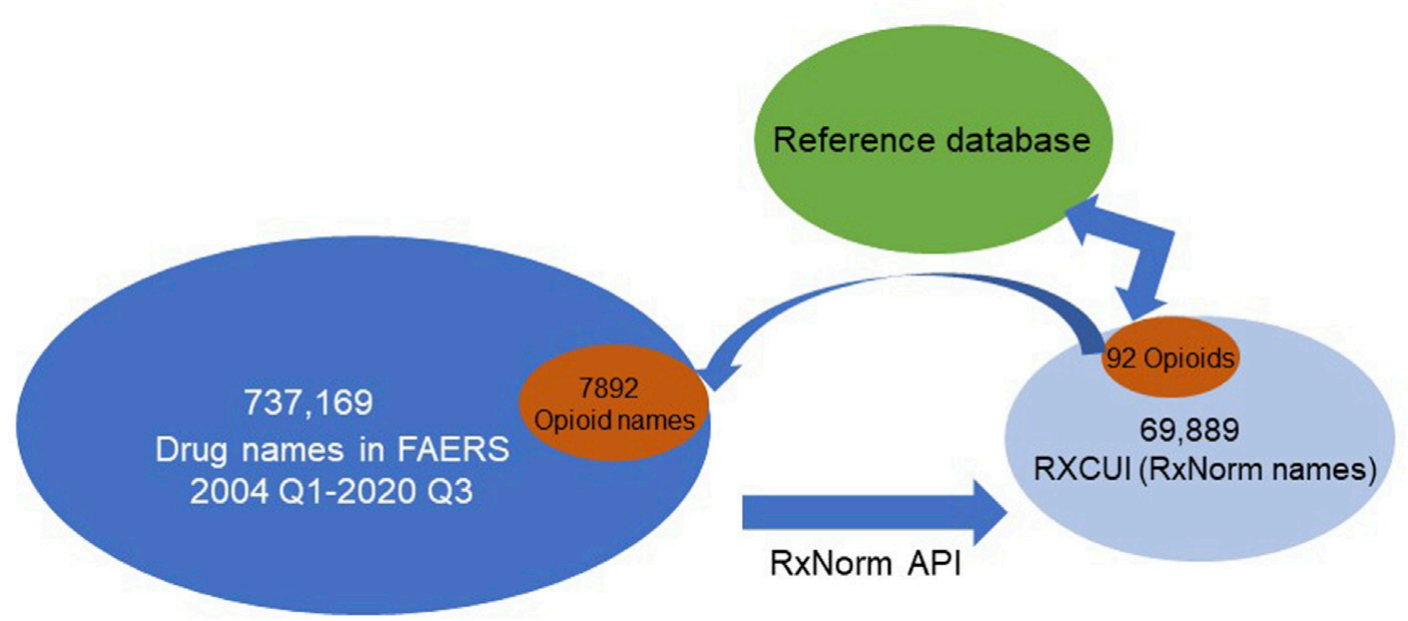
Diagram and results of the case study.

To demonstrate the application and function of the reference database for drug normalization, we have used the prescription opioid entries in FAERS as a case study. After normalizations, we obtained 20,178,515 drug-AE pairs from 2004 Quarter 1 through 2020 Quarter 3. In total, there were 737,169 unique drug names in these reports. After mapping to their equivalent RxCUIs (or RxNorm names), 69,889 unique numbers of RxCUIs (or RxNorm names) were obtained (Figure 2) which was less than 10% of the total drug names in those reports. These unique RxCUIs were matched with those in the reference RxCUI database of FDA-approved opioids and 92 RxCUIs (or RxNorm names) were identified. When we compared this with the dataset of originally retrieved adverse event reports from 2004 Quarter 1 to 2020 Quarter 3, the 92 RxCUIs corresponded with 7,892 different opioid names before RxNorm API mapping. With the application of the reference database, we could easily retrieve the FDA-approved opioid-associated RxCUIs from a big dataset and avoided missing or duplicating information from different formats of drug names. In addition, the fact that 69,889 unique RXCUIs represented the original 737,169 drug names after mapping with RxNorm API revealed that the RxNorm API mapping could greatly (737,169/69,889 = 10.5 times) reduce the usage heterogeneities of drug names by assigning the different drug name with the same active components into the same group (RXCUIs). This was also confirmed by the correspondence of 92 opioids with 7,892 opioid names in the original dataset (7,892/92 = 85.8 times, Figure 2).

**FIGURE 3:**
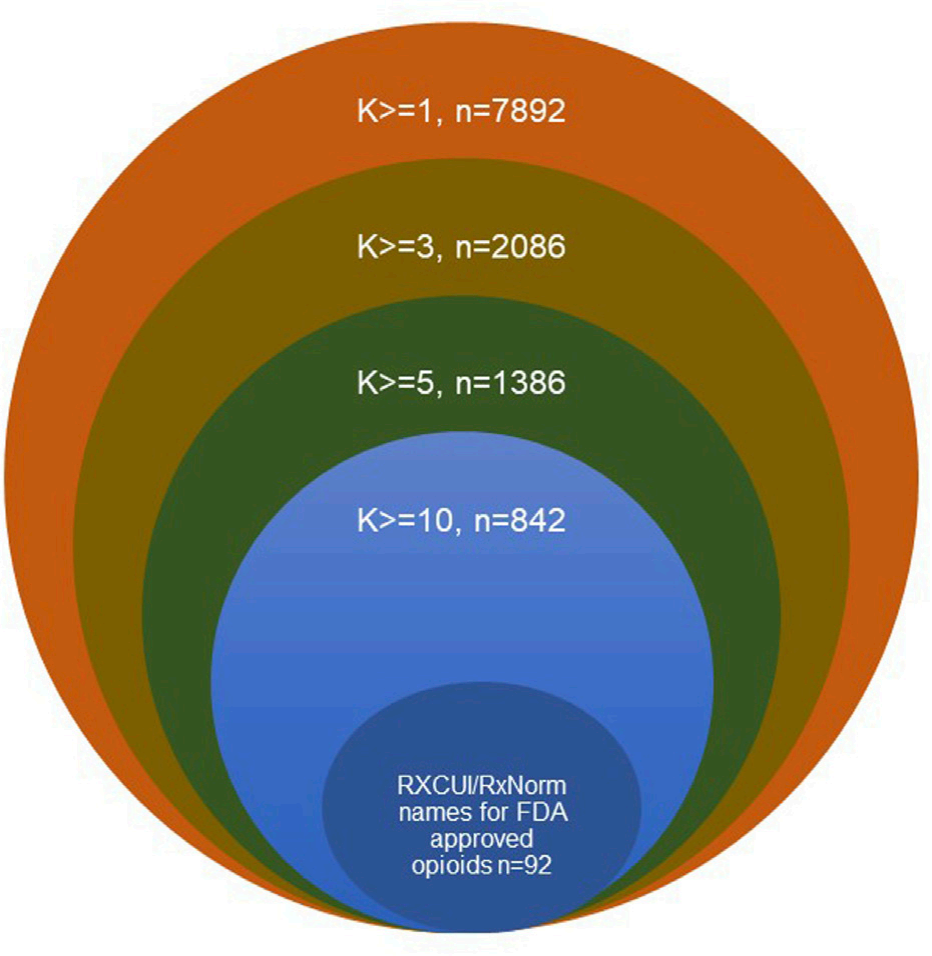
Schematic diagram of usages of different drug names in the dataset.

To further understand the usage status of different drug names, the usage frequencies were calculated for different drug names that were used at least one time (k>=1), three times (k > = 3), five times (k > = 5) and ten times (k > = 10, Table 1; Figure 3). The number of RxNorm names for the FDA-approved opioids (92) and their total usage frequency (936,716) did not change with *k*, while the total number of different drug names and their total usage frequencies decreased when *k* increased. For example, when k > = 1, there were 7,892 opioid names responding to 92 RxCUIs (RxNorm names) of opioids that were used 1,417,126 times total in this dataset; when k > = 10, the total number of different drug names reduced to 842 (∼89.3%), while their total usage frequency was only reduced 12,734 out of 1,417,126 (∼0.9%). These results reveal the large heterogeneities in the usage of opioid names due to the different variations on drug names used by the public. Among the 842 different formats of the drug names that were used at least ten times, the 92 standard RxNorm names contributed around 66.1% (936,716/1,417,126) of the total usage frequencies, the other 750 (842–92) formats of the drug names contributed around 33.0%. If AERs are extracted using the 92 RxNorm names only (i.e., standard names) from FAERS or other adverse events databases, a large amount of information would be missing in the dataset, which may lead to inaccuracy and misinterpretation of the data and its analysis results. The drug name normalization by an appropriate approach is therefore very important. The results of this study demonstrated that using RxNorm mapping could reduce the heterogeneity of opioid names and is necessary to build an intact dataset for analysis by collecting any reports with different drug names which would otherwise be missing.

**TABLE 1:** The status of usages of different opioid names in the dataset.

No. of used frenquences	No. of different drug names	Total used frequencies of different drugs	No. of RxNorm Names	Total used frequencies of RxNorm names
k > = 1	7,892	1,417,126	92	936,716
k > = 3	2,086	1,410,262	92	936,716
k > = 5	1,386	1,407,883	92	936,716
k > = 10	842	1,404,392	92	936,716

To further describe the results in detail, we collected the opioid RxNorm names which had at least two different names and where each name was used at least three times (to exclude the possibility of spelling errors) in the opioid-AE dataset from FAERS and calculated the usage frequencies of each individual name. The data mining results are listed in Table 2. Sixty seven out of 92 RxNorm names of FDA-approved opioids were filtered out and categorized into 13 opioid groups, including buprenorphine, codeine, dihydrocodeine, fentanyl, hydrocodone, hydromorphone, meperidine, methadone, morphine, oxycodone, oxymorphone, tapentadol, and tramadol. Each group has a few opioids with their corresponding RxCUI numbers and RxNorm names identified (Table 2). Each RxNorm drug was reported with several different names, and the numbers of names as well as the usage frequencies were reported. It was observed that more than 37.3% (25 out of 67) of RxNorm names had the percentages of their usage frequencies above 90%, possibly due to the drug commonalities and ease of remembering (e.g., “codeine” or “opana”). Conversely, 14 RxNorm names had a percentage of usage frequency less than 0.1%. It was noticed that these RxNorm names were mostly combined opioids (i.e., opioids with other ingredients such as aspirin or acetaminophen) and in the format of long strings with special characters “/”, for example, “aspirin/oxycodone” or “acetaminophen/caffeine/dihydrocodeine”. Hence, it might be difficult for patients to remember accurately the standard names of these combined opioids leading to entry of different names when reporting their AEs in FAERS. In addition, more than half of these 67 FDA-approved opioids had more than 10 various names other than their RxNorm names, with three of them having even more than 200 various names. They are “acetaminophen/codeine”, “acetaminophen/hydrocodone” and “tramadol hydrochloride”, which have been popularly used for years but potentially could not be remembered and typed correctly due to their long names. For example, in the class of hydrocodone, there were 286 various names under the same RxCUI number 214182 but the standard name (RxNorm name) acetaminophen/hydrocodone was only used 73 times in the dataset, and the rest of the 285 various names were used 77,666 (77,739–73) times, within which three different names “hydrocodone bitartrate and acetaminophen”, “hydrocodone bitartrate/acetaminophen”, and “hydrocodone/acetaminophen” were used more than 10,000 times, respectively (data not shown here). A search using only the RxNorm standard name to retrieve the reports from FAERS would not include more than 99% of the AERs in the dataset, which might result in biased and inaccurate analysis results.

**TABLE 2:** The total number of different drug names and their total usage frequencies.

Opioid classes	RxCUI	RxNorm Name	No. of different names	Usage frequency of RxNorm name	Usage frequency of all different names	Percentage (%) of RxNorm name usage
buprenorphine	1716058	belbuca	6	553	577	95.8
	203840	buprenex	5	57	98	58.2
	1819	buprenorphine	28	2,032	10,383	19.6
	904871	butrans	13	52,880	52,992	99.8
	352364	buprenorphine/naloxone	52	33	2,626	1.3
	352990	suboxone	9	19,420	21,450	90.5
codeine	2670	codeine	45	24,572	25,926	94.8
	817579	acetaminophen/codeine	252	4	28,225	0.0
	993764	capital and codeine	2	12	17	70.6
	993837	acetaminophen 300 mg/codeine phosphate 30 mg oral tablet [tylenol with codeine]	18	0	3,544	0.0
	220586	tylenol with codeine	18	7,948	9,708	81.9
	689561	acetaminophen/butalbital/caffeine/codeine	6	0	73	0.0
	217126	fioricet with codeine	4	271	408	66.4
	214160	aspirin/butalbital/caffeine/codeine	5	0	19	0.0
	217127	fiorinal with codeine	7	156	321	48.6
	689522	aspirin/carisoprodol/codeine	2	0	107	0.0
	214444	codeine/promethazine	20	0	412	0.0
dihydrocodeine	23088	dihydrocodeine	24	2,515	4,183	60.1
	689569	acetaminophen/caffeine/dihydrocodeine	10	0	88	0.0
	746611	trezix	2	28	52	53.8
fentanyl	1053648	abstral	11	959	1,115	86.0
	215008	actiq	2	26,948	26,952	100.0
	151678	duragesic	35	36,144	42,325	85.4
	4337	fentanyl	52	26,441	77,019	34.3
	668619	fentora	10	26,069	27,515	94.7
	1115547	lazanda	5	199	219	90.9
	1237051	subsys	4	9,191	9,372	98.1
	49991	droperidol/fentanyl	5	0	64	0.0
hydrocodone	5489	hydrocodone	30	48,914	51,638	94.7
	1442523	zohydro	3	444	450	98.7
	214182	acetaminophen/hydrocodone	286	73	77,739	0.1
	144254	lortab	31	21,941	23,044	95.2
	491666	lorcet	13	524	1,042	50.3
	218772	norco	22	57,621	57,839	99.6
	128793	vicodin	47	41,950	42,663	98.3
	214614	homatropine/hydrocodone	26	0	479	0.0
	992657	hycodan	12	392	569	68.9
	220542	tussigon	3	14	25	56.0
	214392	chlorpheniramine/hydrocodone	22	0	333	0.0
	730984	tussicaps	3	44	53	83.0
	214627	hydrocodone/ibuprofen	28	131	867	15.1
	220826	vicoprofen	4	1,226	1,239	99.0
	214631	hydrocodone/pseudoephedrine	2	0	15	0.0
hydromorphone	224913	dilaudid	30	50,623	51,177	98.9
	902730	exalgo	6	9,647	9,963	96.8
	3423	hydromorphone	70	42,380	48,255	87.8
meperidine	6754	meperidine	38	901	2,530	35.6
methadone	202370	dolophine	6	476	619	76.9
	218337	methadone hydrochloride	14	1,261	14,717	8.6
	152751	methadose	3	3,079	3,103	99.2
morphine	203240	kadian	9	21,926	22,161	98.9
	30236	morphine sulfate	58	15,814	45,455	34.8
	203354	ms contin	29	36,726	37,588	97.7
	859959	embeda	3	1,325	1,609	82.3
oxycodone	7804	oxycodone	87	72,895	1,23,099	59.2
	218986	oxycontin	48	92,489	99,146	93.3
	214183	acetaminophen/oxycodone	144	19	21,595	0.1
	42844	percocet	41	71,517	76,726	93.2
	214256	aspirin/oxycodone	12	0	110	0.0
oxymorphone	643147	opana	11	28,670	28,768	99.7
	82064	oxymorphone hydrochloride	2	217	18454	1.2
tapentadol	854137	nucynta	3	28,254	28,332	99.7
	787390	tapentadol	7	1,341	1,528	87.8
tramadol	1148479	conzip	3	51	113	45.1
	82110	tramadol hydrochloride	220	4,994	1,28,410	3.9
	220606	ultram	21	17,853	18,120	98.5
	353062	ultracet	19	4,933	5,220	94.5

To describe the method in more detail, we randomly selected two RxCUI numbers and listed all the various names and their usage frequencies in our retrieved dataset. Table 3 shows the example of the opioid with the RxCUI number of 214256. This opioid had 12 different names shown in the FAERS adverse reports including its standard RxNorm name of “aspirin/oxycodone” (highlighted in red). The different names in Table 3 were sorted by usage frequency from high to low, and the most popularly used name was “oxycodone and aspirin” which was used in 55 FAERS reports in our dataset. The RxNorm standard name “aspirin/oxycodone” was used only four times in addition to ten usages of its inverted word format “oxycodone/aspirin”. The same situation was shown in Table 4 where the opioid with the RXCUI number of 218337 was listed as another example. The RxNorm of RxCUI 218337 is “methadone hydrochloride” (highlighted in red) and was used 1,261 times; while the very similar name “methadone hydrochloride.” was shown in 2,117 FAERS reports in the data set. The most used name for this opioid was “methadone hcl” which was reported 11,040 times, far more than the others. In both randomly selected examples, the standard RxNorm names exhibited in less than 10% of the adverse events reports, with “aspirin/oxycodone” as low as 3%. In fact, most of the adverse event reports had various formats of names instead of the standard RxNorm name since FAERS is a spontaneous reporting system. These two randomly selected examples confirmed the existence of the drug name variations and the potentially severe consequences in FAERS and other spontaneous reporting systems.

**TABLE 3:** The various names and their usage frequencies of the opioid with the RxCUI number of 214256 in our retrieved dataset.

	Drug name	Frequency
1	oxycodone and aspirin	55
2	oxycodone/aspirin	10
3	aspirin w/oxycodone	9
4	oxycodone and aspirin/00554201/	5
5	oxycodone/asa	5
6	aspirin/oxycodone	4
7	oxycodone with aspirin	4
8	oxycodone-aspirin	4
9	oxycodone-asa	4
10	oxycod/asa	4
11	aspirin + oxycodone	3
12	oxycodone and asa	3
	Total	110

**TABLE 4:** The various names and their usage frequencies of the opioid with the RxCUI number of 218337 in our retrieved dataset.

	Drug name	Frequency
1	methadone hcl	11,040
2	methadone hydrochloride	2,117
3	methadone hydrochloride	1,261
4	methadone ap hp	215
5	methadone/methadone hydrochloride	17
6	methadone ap?hp	12
7	methadone (chlorhydrate de)	11
8	methadone chlorhydrate ap hp	9
9	ketalgin (methadone)	8
10	methadone [methadone hydrochloride]	8
11	methadon alternova	6
12	methadon hcl	6
13	methadone chlorhydrate	4
14	methadone intensol	3
	Total	14,717

## 4 Conclusion

It is extremely important to recognize and identify variations in drug names when using spontaneous reporting databases for data analysis. In responding to this challenge, it is necessary to explore appropriate methods and algorithms that aim to automatically standardize and link different representations of the same drugs to build an intact and high-quality database for all kind of research, especially during postmarketing data analysis in pharmacovigilance. In addition, drug name normalization is also a crucial step in computerization and machine learning analysis of drug-related data. As a conclusion, in this study, we have developed a workflow to provide a standardized nomenclature for clinical drugs and for drug name normalization by using the RxNorm API system. The case study of prescription opioids analysis of FAERS data confirmed the importance of drug name normalization and the effectiveness and accuracy of the proposed method. The reference opioid/RxNorm/RxCUI database, as a derivative product of the method in the case study, will provide a simple and direct way for drug reviewers and analysts to conduct pharmacovigilance and safety analysis, where AEs and drug interactions are monitored. In addition, the drug names normalized by this method provide consistent and standardized inputs as a foundation for machine learning algorithms and predictive modeling for future prediction and analysis tasks.

## Data Availability

Publicly available datasets were analyzed in this study. This data can be found here: https://www.fda.gov/drugs/surveillance/questions-and-answers-fdas-adverse-event-reporting-system-faers.
